# Robust Behavior Recognition in Intelligent Surveillance Environments

**DOI:** 10.3390/s16071010

**Published:** 2016-06-30

**Authors:** Ganbayar Batchuluun, Yeong Gon Kim, Jong Hyun Kim, Hyung Gil Hong, Kang Ryoung Park

**Affiliations:** Division of Electronics and Electrical Engineering, Dongguk University, 30 Pildong-ro 1-gil, Jung-gu, Seoul 100-715, Korea; ganabata87@dongguk.edu (G.B.); csokyg@dongguk.edu (Y.G.K.); zzingae@naver.com (J.H.K.); hell@dongguk.edu (H.G.H.)

**Keywords:** intelligent surveillance system, visible light camera, thermal camera, behavior recognition

## Abstract

Intelligent surveillance systems have been studied by many researchers. These systems should be operated in both daytime and nighttime, but objects are invisible in images captured by visible light camera during the night. Therefore, near infrared (NIR) cameras, thermal cameras (based on medium-wavelength infrared (MWIR), and long-wavelength infrared (LWIR) light) have been considered for usage during the nighttime as an alternative. Due to the usage during both daytime and nighttime, and the limitation of requiring an additional NIR illuminator (which should illuminate a wide area over a great distance) for NIR cameras during the nighttime, a dual system of visible light and thermal cameras is used in our research, and we propose a new behavior recognition in intelligent surveillance environments. Twelve datasets were compiled by collecting data in various environments, and they were used to obtain experimental results. The recognition accuracy of our method was found to be 97.6%, thereby confirming the ability of our method to outperform previous methods.

## 1. Introduction

Accurate recognition of behavior is very important and a challenging research topic in intelligent surveillance systems. Although this system should be also operated during the nighttime, objects cannot be visualized in images acquired by a conventional visible light camera. Therefore, a near infrared (NIR) camera or thermal camera (based on medium-wavelength infrared (MWIR) or long-wavelength infrared (LWIR) light) has been considered for usage during the nighttime as an alternative.

The NIR camera usually requires an additional NIR illuminator, which should illuminate a wide area over a great distance during nighttime. However, a thermal camera usually does not need an additional illuminator. In addition, an intelligent surveillance system should be operated during both the daytime and nighttime. Considering all these factors, a dual system of visible light and thermal cameras is used in our research, and we propose a new behavior recognition in intelligent surveillance environments.

A thermal camera image clearly reveals the human body at night and in winter by measuring the human body temperature, which is detectable in the range of MWIR of 3–8 μm and LWIR of 8–15 μm [1,2,3,4,5,6,7,8,9,10,11,12]. Based on these characteristics, Ghiass et al. [12] introduced various methods of infrared face recognition, also. However, the distinction between the human and the background in the thermal image diminishes when the background temperature is similar to that of the human in some situations during the daytime, which can reduce the consequent recognition accuracy of behavior. Therefore, the use of both thermal and visible light images enables us to enhance the recognition accuracy of behavior in intelligent surveillance system.

There have been previous studies where the fusion of visible light and thermal cameras is used for various applications [13,14,15,16]. Davis et al. [13] proposed the fusion method for human detection from visible light and thermal cameras based on background modeling by statistical methods. Arandjelović et al. [14] proposed the face recognition method by combining the similarity scores from visible light and thermal camera-based recognition. Leykin et al. [15] proposed the method for tracking pedestrians based on the combined information from visible light and thermal cameras. In addition, Kong et al. [16] proposed the face recognition method robust to illumination variation based on the multiscale fusion of visible light and thermal camera images.

Although there have been previous studies combining visible light and thermal cameras in various applications, we focus on the behavior recognition in this research. Previous research on behavior recognition can be categorized into two groups: single camera-based and multiple camera-based approaches. Rahman et al. [17,18,19,20] proposed the single camera-based approaches which used negative space analysis based on a background region inside the detected box of the human body. However, these approaches deliver limited performance in cases in which there is a large difference between the detected box and the box that was defined based on the actual human area. Fating and Ghotkar [21] used the histogram of the chain code descriptor to analyze the boundary of the detected area. Although the histogram represents the efficient features, it is computationally expensive to obtain the chain codes from all the shapes of the detected area. A Fourier descriptor was used to extract the scale and rotation invariant features for the detected foreground area [22,23,24,25]. This method has the advantage of correctly representing the foreground shape, but it has difficulty in discriminating behavior with a similar foreground shape, such as walking and kicking. Sun et al. [26] proposed the method using a local descriptor based on scale invariant feature transform (SIFT) and holistic features by Zernike moments for human action recognition. However, their method has the disadvantage of requiring much processing time to extract the features by using both the SIFT and Zernike moments. Schüldt and Laptev et al. [27,28,29] performed behavior recognition based on the local spatiotemporal features, although a clear background is required to guarantee highly accurate results. In addition, corner detection-based feature extraction can cause the false detections of corners, which can degrade the accuracy of behavior recognition. Wang et al. and Arandjelović [30,31] proposed the concept of motionlets, a motion saliency method, which achieves high performance in terms of human motion recognition provided the foreground is clearly segmented from the background. Optical flow-based methods were used to represent motion clearly using a feature histogram. However, the process of obtaining optical flow is computationally expensive, and it is also sensitive to noise and illumination changes [27,28,32]. Various groups [32,33,34,35,36,37] have performed behavior recognition based on a gait flow image (GFI), gait energy image (GEI), gait history image (GHI), accumulated motion image (AMI), and motion history image (MHI). These studies focused on obtaining a rough foreground region in which motion occurs, but the attempt to determine the exact path of motion of hands and legs was less satisfactory. Wong et al. [38] proposed a method of faint detection based on conducting a statistical search of the human’s height and width using the images obtained by a thermal camera. Youssef [39] proposed a method based on the convexity defect feature point for human action recognition. This method finds the tip points of hand, leg, and head. However, incorrect points can be detected as a result of an increase in the number of points for large-sized humans surrounded by noise. In addition, this method cannot discriminate the tip point of a hand from that of a leg in cases in which the leg is positioned at a height similar to that of the hand when kicking motion happens. Besides, it is computationally expensive because of the need to calculate the contour, polygon, convex hull, and convexity defect in every frame.

All this research based on images acquired with a single visible camera has the limitation of performance enhancement when a suitable foreground is difficult to be obtained from the image in situations containing extensive shadows, illumination variations, and darkness. In addition, previous research based on thermal cameras was performed in constrained environments such as indoors or without considering situations in which the background temperature is similar to the foreground in hot weather.

Therefore, multiple camera-based approaches were subsequently considered as an alternative. Zhang et al. [40] proposed a method of ethnicity classification based on gait using the fusion of information of GEI with multi-linear principal component analysis (MPCA) obtained from multiple visible light cameras. Rusu et al. [41] proposed a method for human action recognition by using five visible light cameras in combination with a thermal camera, whereas Kim et al. [42] used a visible light camera with a thermal camera. However, all these studies were conducted in indoor environments without considering temperature and light variations associated with outdoor environments. 

We aim to overcome these problems experienced by previous researchers, by proposing a new robust system for recognizing behavior in outdoor environments including various temperature and illumination variations. Compared to previous studies, our research is novel in the following three ways:

- Contrary to previous research where the camera is installed at the height of a human, we research behavior recognition in environments in which surveillance cameras are typically used, i.e., where the camera is installed at a height much higher than that of a human. In this case, behavior recognition usually becomes more difficult because the camera looks down on humans.
- We overcome the disadvantages of previous solutions of behavior recognition based on GEI and the region-based method by proposing the projection-based distance (PbD) method based on a binarized human blob.
- Based on the PbD method, we detect the tip positions of the hand and leg of the human blob. Then, various types of behavior are recognized based on the tracking information of these tip positions and our decision rules.

The remainder of our paper is structured as follows. In Section 2, we describe the proposed system and the method used for behavior recognition. The experimental results with analyses are presented in Section 3. Finally, the paper is concluded in Section 4.

## 2. Proposed System and Method for Behavior Recognition

### 2.1. System Setup and Human Detection

In this section, we present a brief introduction of our human detection system because the behavior recognition is carried out based on the detection results.

**Figure 1:**
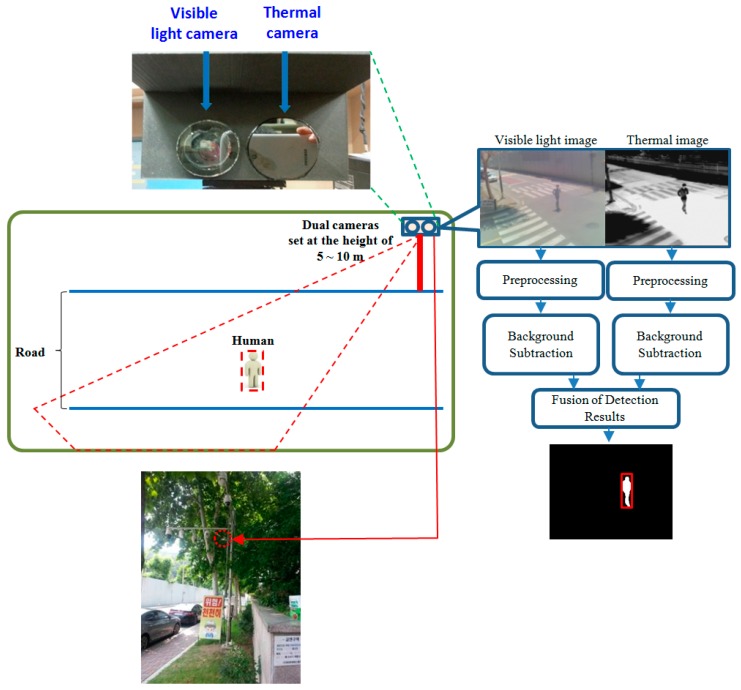
Examples of system setup with brief explanations of our human detection method.

Figure 1 shows the examples of our system setup and human detection method. Detailed explanations of the system and our human detection method can be found in our previous paper [43]. We have implemented dual camera systems (including an FLIR thermal camera capturing the images of 640 × 480 pixels [44] and a visible light camera capturing the images of 800 × 600 pixels), where the two axes of the visible light and thermal cameras are oriented parallel to the horizontal direction as shown in the upper left image of Figure 1. Therefore, the visible light and thermal images are simultaneously captured with minimum disparity. We prevented rain from entering the thermal and visible light cameras by attaching a glass cover (germanium glass, which is transparent to MWIR and LWIR light [45] and conventional transparent glass for the thermal and visible light cameras, respectively) to the front of each camera, as shown in the upper left image of Figure 1. Our dual camera system is set at a height of 5–10 m above the ground, as shown in Figure 1. In addition, we tested it at five different heights (see details in Section 3.1) to measure the performance of our system in various environments.

The flow diagram on the right-hand side of Figure 1 summarizes our human detection algorithm. Through background subtraction after preprocessing to remove noise, two human regions are detected from the visible light and thermal images. Then, the two regions are combined based on a geometric transform matrix, which is obtained by the calibration procedure of both cameras in advance. Finally, the correct human area is detected as shown on the right of Figure 1 after post-processing for noise reduction, morphological operation, and size filtering [43,46]. Our detection method can be applied to images including multiple humans. The use of dual camera systems enables our method to robustly detect human areas in the images of various environments such as those with severe shadow, illumination variations, darkness, and cases in which the background temperature is similar to human area in hot weather.

**Figure 2:**
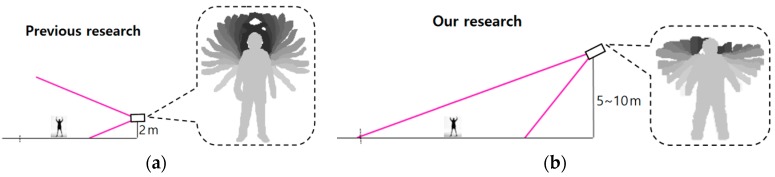
Comparison of different camera setup used in (**a**) previous research; (**b**) our research.

Most previous research on behavior recognition was performed with images captured by a camera at low height. That is, the camera was set at a height of about 2 m above the ground and can capture frontal-viewing human body parts as shown in Figure 2a. This arrangement allowed the motions of human hands and feet to be observed more distinctively. However, our camera is set at a height of 5–10 m, which is the normal height of the camera of a surveillance system. This causes the human area in the image to shrink in the vertical direction as shown in Figure 2b. In addition, our camera arrangement could also reduce the motions of human hands and feet in the vertical direction, change the ratio of height to width of the human box, and reduce the vertical image resolution of the human box in the image, all of which can complicate the correct recognition of behavior. However, our research needs to consider that our system is intended for use in a conventional surveillance system; therefore, the camera setup shown in Figure 2b is used in our research, and we extract the tip points of the hand and leg to track them for accurate behavior recognition rather than using the entire region covering the detected human body.

### 2.2. Proposed Method of Behavior Recognition

#### 2.2.1. Overall Procedure of Behavior Recognition

**Figure 3:**
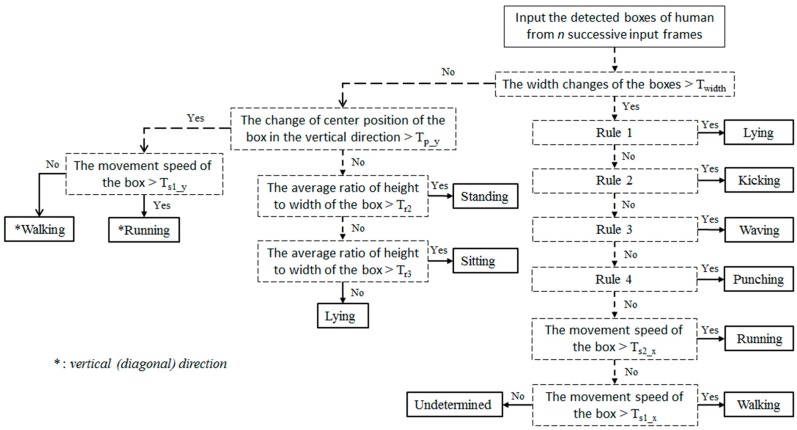
Flowchart of the proposed method of behavior recognition.

The method proposed for behavior recognition is presented in Figure 3. Detailed explanations of Rules 1–4 are provided in Section 2.2.2. We adopted an IF-THEN rule-based method to recognize behavior. As shown in Figure 3, behaviors are roughly divided into two types according to the horizontal activeness measured by the change in the width of detected human box. Our research aims to recognize the following 11 types of behavior:

- Waving with two hands
- Waving with one hand
- Punching
- Kicking
- Lying down
- Walking (horizontally, vertically, or diagonally)
- Running (horizontally, vertically, or diagonally)
- Standing
- Sitting
- Leaving
- Approaching

Among the 11 types of behavior, leaving and approaching are recognized simply based on the change in the distance between the two center positions of the detected boxes of two people. If the distance is reduced, our system determines it as approaching, whereas if it increases, our method determines it as leaving.

Behavior such as hand waving, punching, and kicking has very complicated motion patterns. For example, waving can either be full waving or half waving. We assume the directions of 3, 6, 9, and 12 o’clock to be 0°, 270°, 180°, and 90°, respectively.

**Figure 4:**
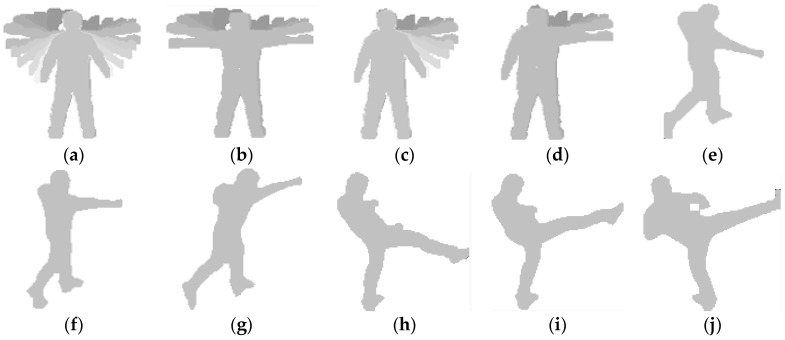
Comparison of motion patterns of each type of behavior. Waving: (**a**) full, by using two hands; (**b**) half, by using two hands; (**c**) full, by using one hand; (**d**) half, by using one hand. Punching: (**e**) low; (**f**) middle; (**g**) high. Kicking: (**h**) low; (**i**) middle; (**j**) high.

- Full waving, where the arm starts from about 270° and moves up to 90° in a circle as shown in Figure 4a,c.
- Half waving, where the arm starts from about 0° and moves up to 90° in a circle as shown in Figure 4b,d.
- Low punching, in which the hand moves directly toward the lower positions of the target’s chest shown in Figure 4e.
- Middle punching, where the hand moves directly toward the target’s chest as shown in Figure 4f.
- High punching, where the hand moves directly toward the upper positions of the target’s chest as shown in Figure 4g.
- Similarly, in low, middle, and high kicking, the leg moves to different heights as shown in Figure 4h–j, respectively.

In addition, according to martial arts, kicking and punching can be shown in many different ways.

In our research, the 11 types of behavior are categorized into three classes, with each class representing behavior with different intention and meaning. This requires us to extract the different features according to each class.

*Class 1*: **Walking**, **running**, **sitting**, and **standing** are regular types of behavior with less detailed gestures of the hands and feet than the behavior in Class 2. Walking and running behavior involves active motion, whereas sitting and standing behavior is motionless. In addition, the rough shape of the human body when standing is different from that when sitting. Therefore, the motion and rough shape of the human body are measured using the speed and ratio of height to width of the detected human box, respectively. That is, the speed and ratio are key features of the spatial and temporal information of the detected box that enable recognition of the regular types of behavior of Class 1.

*Class 2*: **Kicking**, **punching**, **lying down**, **waving with two hands**, and **waving with one hand** are types of behavior consisting of special gestures for hands or feet, motion, and shape except for lying down. In addition, they are characterized by very active motions of the hands or feet. Some of the behavior produces similar shapes in an image, such as waving with one hand and punching. Therefore, we need to be able to track feature points efficiently, which we achieve by using the proposed PbD method to find the tip points of legs and hands as feature points. We track the path of these tip points to recognize the behavior in this class. However, lying down can be recognized in the same way as the behavior in Class 1, because it is motionless and the ratio of height to width of the human box is different from that of other behaviors.

*Class 3*: **Approaching** and **leaving** are interactional types of behavior, both of which are recognized by measuring the change in the distance between the boxes of two persons. 

#### 2.2.2. Recognition of Behavior in Classes 1 and 3

**Figure 5:**
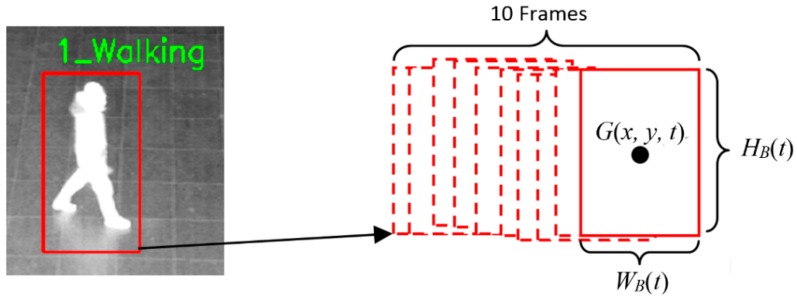
Example showing the parameters used to analyze the detected human box by comparing the box in the current frame with those in the previous nine frames.

In this section, we explain the proposed method in terms of the recognition of behavior in Classes 1 and 3. Behavior in Classes 1 and 3 is recognized by analyzing and comparing the detected human box in the current frame with the previous nine boxes from the previous nine frames as shown in Figure 5.

As shown in Equation (1), the sum of change in the width of detected box is calculated from 10 frames (current and previous nine frames):

(1) $$W_{v}=\overset{N-1}{\underset{i=0}{\sum }}\left|W_{B}\left(t-\left(i+1\right)\right)-W_{B}\left(t-i\right)\right|\;$$

where *N* is 9 and $W_{B}\left(t\right)$ is the width of the detected human box at the *t-*th frame as shown in Figure 5. Based on $W_{v}$ compared to the threshold (T_width_), the first classification for behavior recognition is conducted as shown in Figure 3.

If $W_{v}$ is less than the threshold (T_width_), the sum of change in the center of the detected box in the vertical direction is calculated from 10 frames (current and previous nine frames). Based on the sum compared to the threshold (T_p_y_), walking and running behaviors in the vertical or diagonal direction are distinguished from those of standing, sitting, and lying down as shown in the left-hand part of Figure 3.

The distance between the center positions (*G*(*x, y, t*) of Figure 5) of two detected boxes is measured by comparing two successive frames among the 10 frames (current and previous nine frames). Then, the consequent movement velocity of the center position can be calculated as the movement speed of the detected box because the time difference between two successive frames can be obtained by our system. Based on the average speed (considering the movement direction) calculated from 10 frames (current and previous nine frames), walking and running are recognized. If the speed exceeds the specified threshold, our system determines the behavior as running, otherwise the behavior is determined as walking, as shown in Figure 3. 

In general, the distance between two center positions in the image in the horizontal direction is different from that in the vertical or diagonal directions even with the same distance in 3D space. That is because the camera setup is tilted as shown in Figure 2b. Therefore, a different threshold (T_s1_y_) is used for recognizing walking and running in the vertical or diagonal directions compared to the thresholds (T_s1_x_ and T_s2_x_) for the horizontal direction, as shown in Figure 3.

The ratio of the height to width of the detected box is calculated by Equation (2), and based on the average *R* from 10 frames (current and previous nine frames), standing, sitting, or lying down are recognized. As shown in Figure 3, if the average *R* is larger than the threshold (T_r2_), our system determines the behavior as standing. If the average *R* is less than threshold (T_r2_), but larger than threshold (T_r3_), the behavior is recognized as sitting. If the average *R* is less than both threshold (T_r2_) and threshold (T_r3_), our system determines the behavior as lying down.

(2) $$R=\frac{H_{B}\left(t\right)}{W_{B}\left(t\right)}$$

where $W_{B}\left(t\right)$ and $H_{B}\left(t\right)\;$represent the width and height, respectively, of the detected human box at *t-*th frame as shown in Figure 5.

Approaching and leaving behavior is recognized by measuring the change in the distance between the center positions of the boxes of two persons, as shown in Equation (3):

(3) $$D_{v}=\overset{N-1}{\underset{i=0}{\sum }}\left(b\left(t-\left(i+1\right)\right)-b\left(t-i\right)\right)$$

where *N* is 9 and *b*(*t*) is the Euclidean distance between the two center positions of the boxes of two persons at the *t-*th frame. If $D_{v}$ is larger than the threshold, the behavior is determined as approaching, whereas the behavior is determined as leaving if $D_{v}$ is smaller than the threshold.

The optimal thresholds in the flowchart of Figure 3 were experimentally determined to minimize the error rate of behavior recognition. To ensure fairness, the images used for the determination of the thresholds were excluded from the experiments that were conducted to measure the performance of our system.

#### 2.2.3. Recognition of Behavior in Class 2

**Figure 6:**
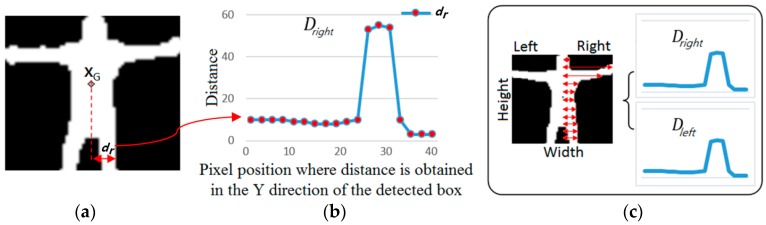
Example of profile graphs produced by the proposed PbD method. (**a**) Binarized image of human area in the detected box; (**b**) profile graph representing the right part of the human area; (**c**) two profile graphs representing the right and left parts of the human area, respectively.

We developed a new method, named the PbD method, to extract the features of behavior in Class 2. Firstly, *X_G_* is calculated as the geometric center position of the binarized image of the human area as shown in Figure 6a. Then, the distances (*d_r_* (or *d_l_*)) between the X positions of *X_G_* and the right-most (or left-most) white pixel of the human area are calculated at each Y position of the detected human box. The processing time was reduced by calculating the distances at each Y position for every three pixels. These distances enable us to obtain the two profile graphs ($D_{left}\text{ and }D_{right}$) including the leg, body, arm, and head of human area as shown in Figure 6b,c. Because the profile graph is obtained based on the projection of the distance in the horizontal direction, we named this method the PbD method.

(4) $$D_{left}=\left\{d_{l1},d_{l2}\ldots d_{ln}\right\},\;D_{right}=\left\{d_{r1},d_{r2}\ldots d_{rn}\right\}$$

where *n* is the number of distance.

**Figure 7:**
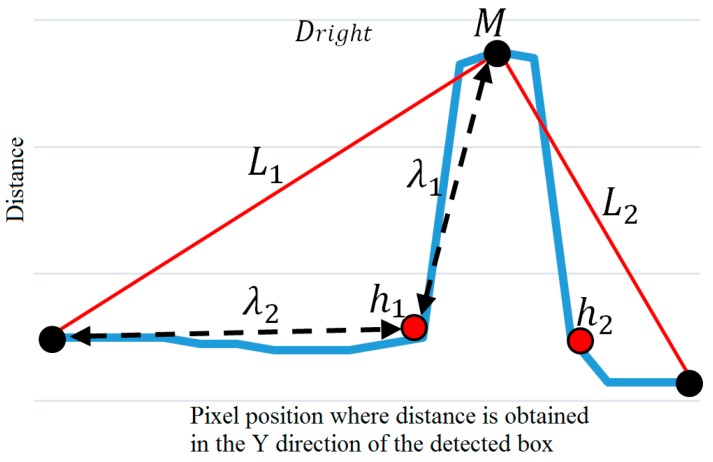
Example illustrating the extraction of features from the profile graph obtained by the proposed PbD method.

The profile graph is used to determine the position whose distance (Y-axis value) is maximized as the tip position (*M* in Figure 7) of the hand or leg. The tip point is determined to either belong to the hand or the leg by obtaining the *L_1_* and *L_2_* lines from the first and last points of the profile graph with point *M* as shown in Figure 7.

Then, the two points on the graph (whose distances from the *L_1_* and *L_2_* lines are maximized, respectively) are detected as the starting and ending points of the human arm, respectively, as shown in *h*_1_ and *h*_2_ of Figure 7. In case the distance from the *L_2_* line is smaller than the threshold, the ending point of the profile graph is determined as *h*_2_. In addition, if the distance from the *L_1_* line is smaller than the threshold, the starting point of the profile graph is determined as *h*_1_.

**Figure 8:**
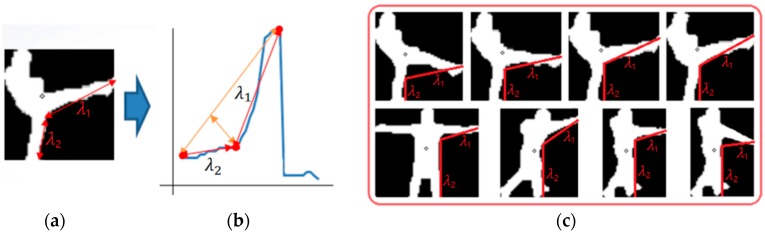
Examples of the two features $\lambda_{1}$ and $\lambda_{2}$ for kicking, and the comparison of these two features for kicking and punching. (**a**) Case of kicking; (**b**) profile graph of (a) by the proposed PbD method; (**c**) comparison of $\lambda_{1}$ and $\lambda_{2}\;$ for kicking and punching.

Based on the starting point, *h*_1_, and the *M* points, the two distances $\lambda_{1}$ and $\lambda_{2}$ can be calculated. Because the human arm is shorter than the distance between the starting position of the arm (*h*_1_ in Figure 7) and the foot (the starting position of the profile graph in Figure 7), $\lambda_{1}$ inevitably becomes smaller than $\lambda_{2}$ when point *M* is the tip position of the hand. On the other hand, because the human leg is longer than the distance between the starting position of the leg and foot, $\lambda_{1}$ inevitably becomes larger than $\lambda_{2}$ when point *M* is the tip position of the leg (foot) as shown in Figure 8b. Based on these observations, we can discriminate between the position of the hand tip and that of the leg tip based on the ratio of $\lambda_{1}$ and $\lambda_{2}$ as shown in Equation (5):

(5) $$\{\begin{array}{c} M\;\mathrm{belongs}\;\mathrm{to}\;\mathrm{hand}\;\mathrm{tip},\;if\;\frac{\lambda_{2}}{\lambda_{1}}>1\\ M\;\mathrm{belongs}\;\mathrm{to}\;\mathrm{leg}\;\mathrm{tip},\;else\; \end{array}$$

**Figure 9:**
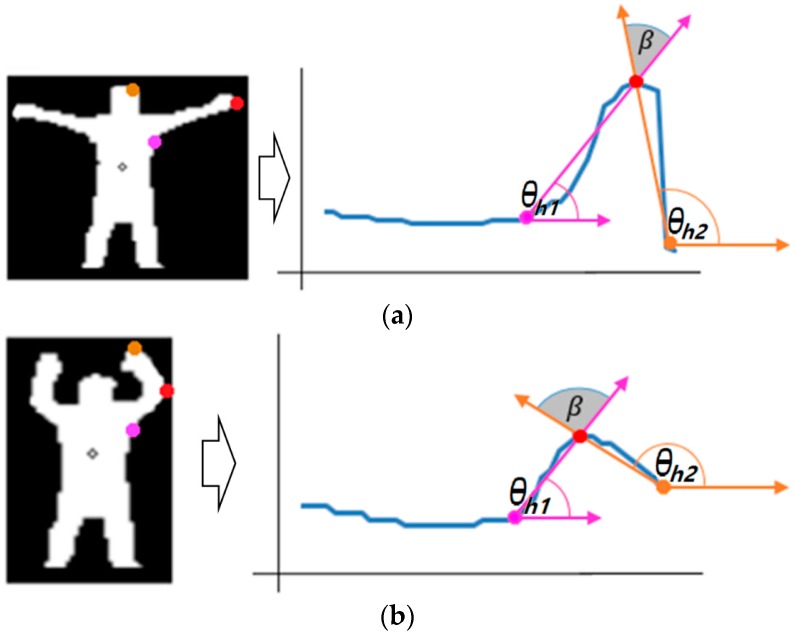
Examples of two types of hand waving by (**a**) unfolding; and (**b**) bending of the user’s arm.

As shown in Figure 9, hand waving behavior can happen by unfolding (Figure 9a) or bending (Figure 9b) the user’s arms. In the latter case (Figure 9b), point *M* can be the tip position of the elbow instead of that of the hand. We proposed the method expressed by Equations (6) and (7) to discriminate the former case (point *M* is the tip position of the hand) from the latter (point *M* is the tip position of the elbow).

As shown in Figure 9, the two angles $\theta_{h1}$ and $\theta_{h2}$ are calculated based on three points of the starting point of the arm, point *M*, and the ending point of the profile graph. Then, the difference between these two angles is calculated as β. In general, β is smaller when hand waving occurs by unfolding than when hand waving occurs by bending of the user’s arm as shown in Figure 9. Therefore, based on β, the former case (point *M* is the tip position of the hand) is discriminated from the latter case (point *M* is the tip position of the elbow) as follows:

(6) $$\{\begin{array}{c} M\;\mathrm{belongs}\;\mathrm{to}\;\mathrm{hand}\;\mathrm{tip},\;if\;\beta <Th_{\theta }\\ M\;\mathrm{belongs}\;\mathrm{to}\;\mathrm{elbow}\;\mathrm{tip},\;else\; \end{array}$$

(7) $$\beta =\text{ abs}(\;\frac{\pi -arc\sin\left(\frac{Y_{M}-Y_{h_{2}}\;}{\sqrt{{\left(X_{M}-X_{h_{2}}\right)}^{2}+{\left(Y_{M}-Y_{h_{2}}\right)}^{2}}}\right)-\;arc\sin\left(\frac{{\left(Y_{M}-Y_{h_{1}}\right)}^{\;}}{\sqrt{{\left(X_{M}-X_{h_{1}}\right)}^{2}+{\left(Y_{M}-Y_{h_{1}}\right)}^{2}\;}}\right)}{\pi /180})$$

where (*X_M_*, *Y_M_*) is the coordinate of point *M* in Figure 7, and ($X_{h_{1}}$, $Y_{h_{1}}$) is the position of *h*_1_. In addition, ($X_{h_{2}}$, $Y_{h_{2}}$) is that of *h*_2_ in Figure 7. *abs* is the function for obtaining absolute value (non-negative value). Based on the extracted feature points of Figure 6, Figure 7, Figure 8 and Figure 9, the types of behavior in Class 2 are recognized based on the decision rules of Table 1. Rules 1–4 of Figure 3 are shown in Table 1.

The proposed PbD method enables us to rapidly find the correct positions of the tips of the hand and leg without using a complicated algorithm that performs boundary tracking of binarized human area.

## 3. Experimental Results

### 3.1. Description of Database

Open databases exist for behavior recognition of visible light images [27,47,48] or that of thermal images [49]. However, there is no open database (for behavior recognition in outdoor environments) with images collected by both visible light and thermal cameras at the same time. Therefore, we used the database that was built by acquiring images with our dual camera system. Data acquisition for the experiments was performed by using a laptop computer and the dual cameras shown in Figure 1. All the images were acquired based on the simultaneous use of visible light and thermal cameras. The laptop computer was equipped with a 2.50 GHz CPU (Intel (R) Core (TM) i5-2520M, Intel Corp., Santa Clara, CA, USA) and 4 GB RAM. The proposed algorithm was implemented using a C++ program using Microsoft foundation class (MFC, Microsoft Corp., Redmond, DC, USA) and OpenCV library (version 2.3.1, Intel Corp., Santa Clara, CA, USA).

We built large datasets that include 11 different types of behavior (explained in Section 2.2.1) as shown in Table 2. Datasets were collected in six different places during the day and at night in environments with various temperatures of four seasons with the different camera setup positions. The database includes both thermal and visible images. The total number of images in the database is 194,094. The size of the human area varies from 28 to 117 pixels in width and from 50 to 265 pixels in height, respectively.

**Figure 12:**
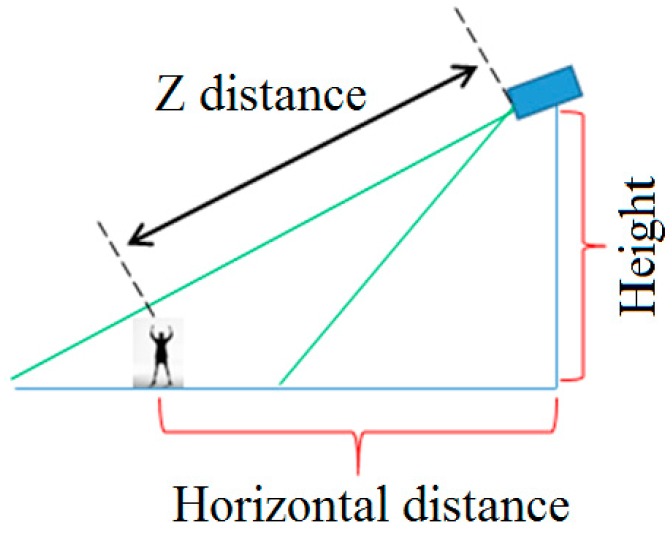
Example of camera setup.

As shown in Figure 12 and Table 3, we collected the 11 datasets with a camera setup of various heights, horizontal distances, and Z distances. We then classified the datasets according to the kind of behavior, again, and the numbers of frames and types of behavior are shown in Table 4.

### 3.2. Accuracies of Behavior Recognition

We used the datasets in Table 4 to evaluate the accuracy of behavior recognition by our method. The accuracies were measured based on the following equations [50]:

(8) $$\text{Positive predictive value }\left(\text{PPV}\right)=\frac{\#\text{TP}}{\#\text{TP}+\#\text{FP}}$$

(9) $$\text{True positive rate }\left(\text{TPR}\right)=\frac{\#\text{TP}}{\#\text{TP}+\#\text{FN}}$$

(10) $$\text{Accuracy}\left(\text{ACC}\right)=\frac{\#\text{TP}+\#\text{TN}}{\#\text{TP}+\#\text{TN}+\#\text{FP}+\#\text{FN}}$$

(11) $$\text{F\_score}=2\cdot \frac{\text{PPV}\cdot \text{TPR}}{\text{PPV}+\text{TPR}}\;$$

where #TP is the number of true positives (TPs), and TP represents the cases in which the behavior included in input image is correctly recognized. #TN is the number of true negatives (TNs), and TN represents the cases in which the behavior not included in input image is correctly unrecognized. In addition, #FP is the number of false positives (FPs), and FP represents the case in which the behavior not included in input image is incorrectly recognized. #FN is the number of false negatives (FNs), and FN represents the case in which the behavior included in input image is incorrectly unrecognized. For all the measures of PPV, TPR, ACC, and F_score, the maximum and minimum values are 100(%) and 0(%), respectively, where 100(%) and 0(%) are the highest and lowest accuracies, respectively.

**Figure 13:**
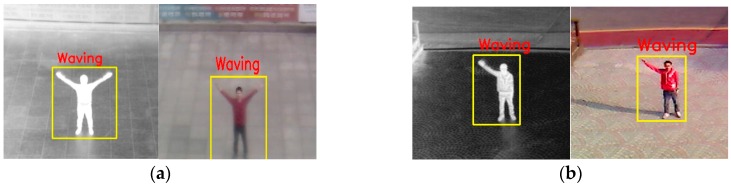

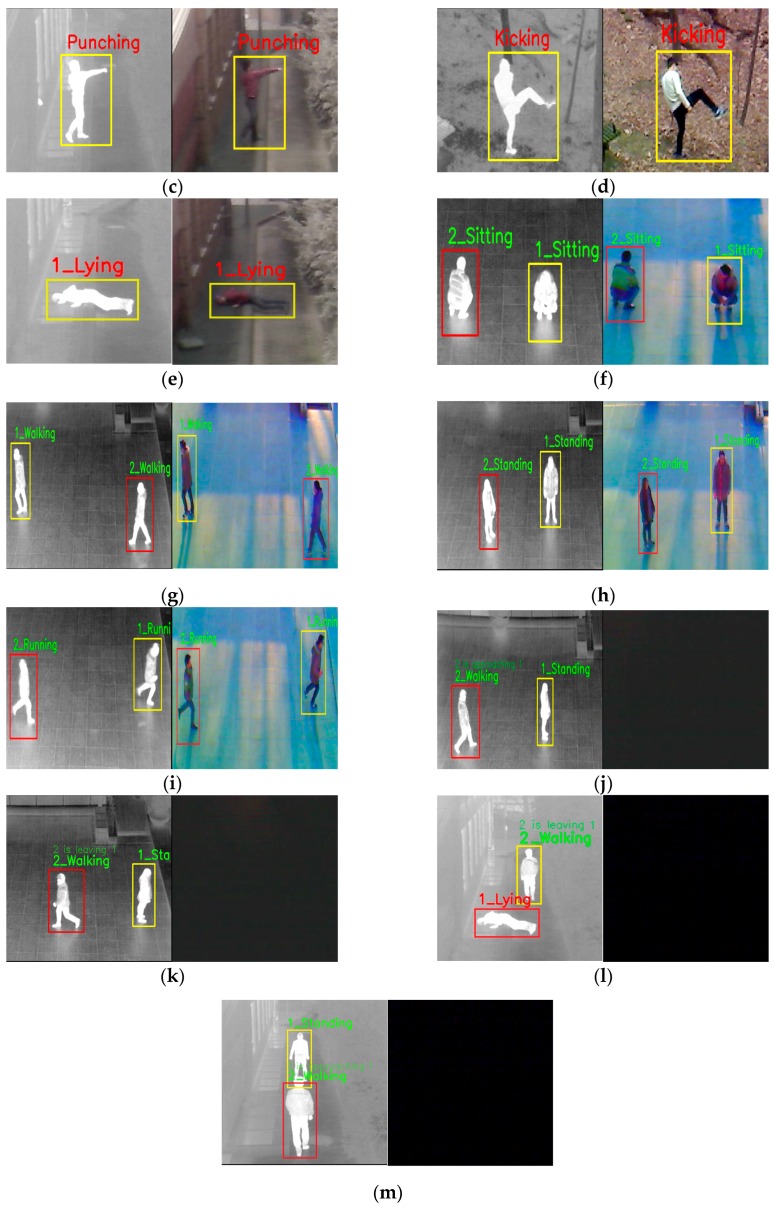
Examples of correct behavior recognition. In (**a**–**m**), the images on the left and right are obtained by thermal and visible light camera, respectively. (**a**) Waving with two hands; (**b**) waving with one hand; (**c**) punching; (**d**) kicking; (**e**) lying down; (**f**) sitting; (**g**) walking; (**h**) standing; (**i**) running; (**j**) and (**m**) approaching (nighttime); (**k**) and (**l**) leaving (nighttime).

As shown in Table 5, the accuracies of behavior recognition by our method are higher than 95% in most cases, irrespective of day and night and kinds of behaviors. In Figure 13, we show examples of correct behavior recognition with our datasets collected in various environments. The results of behavior recognition can show various information of emergency situation. For example, in Figure 13l, one human (object 2) is leaving the other human (object 1), who is lying on the ground, in which case an emergency situation would be suspected.

In the next experiment, we measured the processing time of our method. The procedure of human detection shown in Figure 1 took an average processing time of about 27.3 ms/frame (about 27.7 ms/frame for day images and about 26.9 ms/frame for night images). With the detected human area, the average processing time of behavior recognition of Figure 3 is shown in Table 6. Based on these results, we can confirm that our system can be operated at a speed of about 33.7 frames/s (1000/29.7) including the procedures of human detection and behavior recognition. Considering only the procedure of behavior recognition, this procedure can be operated at a speed of 416.7 (1000/2.4) frames/s.

### 3.3. Comparison between the Accuracies Obtained by Our Method and Those Obtained by Previous Methods

In the next experiment, we performed comparisons between the accuracies of behavior recognition by our method and those by previous methods. These comparisons tested the following methods: Fourier descriptor-based method by Tahir et al. [22], GEI-based method by Chunli et al. [33], and convexity defect-based methods by Youssef [39]. As shown in Table 5 and Table 7, our method outperforms the previous methods for datasets of all types of behavior.

In Table 7, because all the previous methods are able to recognize individual types of behavior (rather than interaction-based behavior), interaction-based behaviors such as leaving and approaching were not compared. The reason for the difference between the ACC and F_score in some cases (for example, punching, by the Fourier descriptor-based method) is that #TN is large in these cases, which increases the ACC as shown in Equation (10).

Since the GEI-based method is based on human motion, motionless behaviors, such as standing, sitting, and lying down, cannot be recognized. The convexity defect-based method is based on a defect point, which cannot be obtained when the distance between two hands and two feet is small. In the case of standing, sitting, and lying down, the distance becomes small; hence, the defect points are not produced. Therefore, these types of behavior cannot be recognized and the recognition results are indicated as “n/a”.

**Figure 14:**
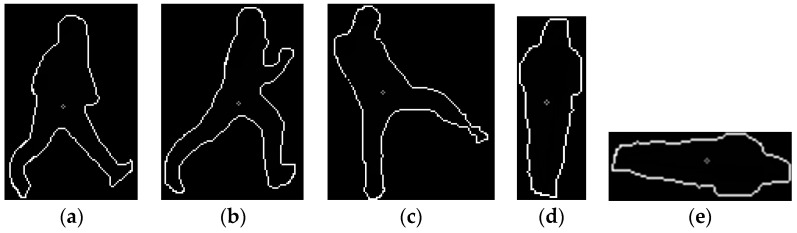
Examples of cases in which the Fourier descriptor-based method produced an erroneous recognition result. (**a**) Walking; (**b**) running; (**c**) kicking; (**d**) standing; (**e**) lying down.

In Fourier descriptor-based methods, walking and lying down are recognized with very low accuracy. This is because walking, running, and kicking behaviors have a very similar pattern of the Fourier descriptor, which means that walking is recognized as kicking or running. Moreover, standing and lying down have similar patterns, such that lying down has been recognized as a standing behavior (see Figure 14a–c). In addition, because of the rotation in variant characteristics of the Fourier descriptor, the Fourier descriptor of the standing behavior in Figure 14d has a pattern similar to that of the lying down behavior in Figure 14e.

**Figure 15:**
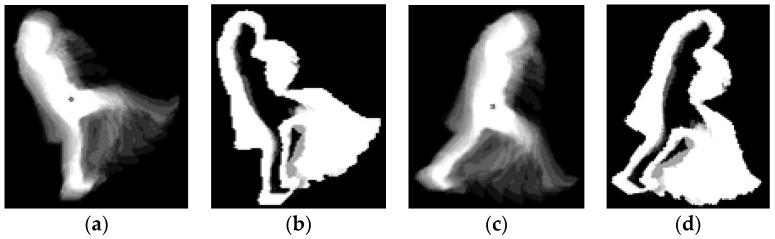
Examples of cases in which the GEI-based method produced an erroneous recognition result. Kicking images by (**a**) GEI; and (**b**) EGEI; running or walking image by (**c**) GEI; and (**d**) EGEI.

GEI-based methods have similar patterns for walking, running, and kicking behaior. Therefore, running was recognized as walking (see Figure 15c,d). In addition, a kicking image produced by GEI is not much different from those showing walking or running as shown in Figure 15. Therefore, the PPV and consequent F_score of kicking are low as shown in Table 7.

**Figure 16:**
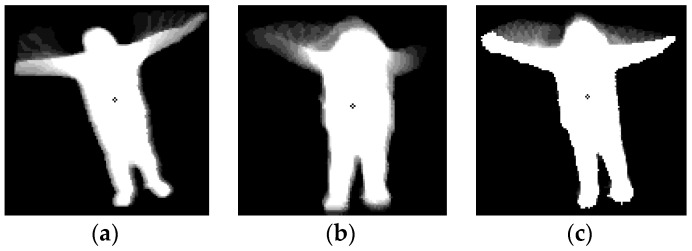
Examples of cases in which the GEI-based method produced an erroneous recognition result. (**a**) A small amount of waving; (**b**,**c**) cases in which information produced by GEI is not distinctive because the image is captured by our camera system installed at a height of 5–10 m (see Figure 12 and Table 3).

In the case of a small amount of hand waving (Figure 16a), GEI-based methods do not provide correct information for behavior recognition. In addition, because our camera set captures images from an elevated position (height of 5–10 m) (see Figure 12 and Figure 16, and Table 3), the information produced by GEI-based methods in the case of hand waving is not distinctive, as shown in Figure 16b,c. Therefore, the accuracies obtained for images showing waving with one or two hands by GEI-based methods are low, as shown in Table 7.

**Figure 17:**
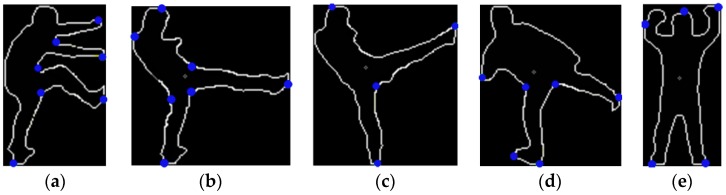
Examples of cases in which the convexity defect-based method produced an erroneous recognition result. (**a**–**d**) Kicking; (**e**) waving with two hands.

The convexity defect-based method produces the errors for behavior recognition of waving, punching, and kicking because of the error of detection of the positions of the hand and leg tip. As shown in Figure 17, in addition to those of the hand and leg tip, other points are incorrectly detected by the convexity defect-based method, which causes an error of behavior recognition for waving, punching, and kicking.

Table 8 presents the behavior recognition results of our method as a confusion matrix. In this table, the actual behavior means the ground-truth behavior, whereas predicted behavior represents that recognized by our method. Therefore, the higher values shown in the diagonal cell of the table (i.e., “standing” both in terms of actual and predicted behaviors) indicate the higher accuracies of behavior recognition. In some cases in Table 8, the sum of values in a row is not 100%, which means that an “undetermined case” scenario, as shown in Figure 3, occurs. For example, for Table 8, in the case of “punching”, the percentage of “undetermined cases” is 15.3% (100 − (0.3 + 1.6 + 0.3 + 82.5)) (%).

In Table 9, we present a summarized comparison of the accuracies and processing time obtained by previous methods and by our method. The processing time only represents the time taken by behavior recognition, and excludes the time for detection of the human box because this is the same in each method. Based on the results in Table 9, we can confirm that our method outperforms other methods both in terms of accuracy and processing time.

Our method is mainly focused on behavioral recognition based on frontal-viewing human body, like most previous studies [17,18,19,20,26,27,30,32,33,34,35,36,38,39,41,42,51]. The change of view direction (from frontal view to side view) does not affect the recognition accuracy of “standing” and “sitting” of Figure 3. Because our method already considers “walking” and “running” in various directions (horizontal, vertical, or diagonal), as shown in Figure 3, the change of view direction does not have much effect on the recognition accuracy of “walking” and “running” in Figure 3, either. However, “lying down” is mainly recognized based on the average ratio of height to width of the detected human box, as shown in Figure 3. Therefore, the recognition accuracy can be highly affected by a change in view direction because the average ratio of height to width of the detected box is changed according to the change of view direction. In the case of behaviors such as “kicking”, “waving”, and “punching”, they are recognized based on the detected tip positions of hands and feet. Therefore, the movement of these positions is difficult to detect in side-viewing images, and the recognition accuracy can be highly affected by the change of view direction. The recognition of “lying down”, “kicking”, waving”, and “punching” only by one side-viewing camera is s challenging problem that has not been solved in previous studies. Therefore, our method is also focused on behavioral recognition based on a frontal view of the human body, like most previous studies.

## 4. Conclusions

In this research, we propose a robust system for recognizing behavior by fusing data captured by thermal and visible light camera sensors. We performed behavior recognition in an environment in which a surveillance camera would typically be used, i.e., where the camera is installed at a position elevated in relation to that of the human. In this case, behavior recognition usually becomes more difficult because the camera looks down on people. In order to solve the shortcomings of previous studies of behavior recognition based on GEI and a region-based method, we propose the PbD method. The PbD method employs a binarized human blob, which it uses to detect the tip positions of hands and legs. Then, various types of behavior are recognized based on the information obtained by tracking these tip positions and our decision rules. We constructed multiple datasets collected in various environments, and compared the accuracies and processing time obtained by our method to those obtained by other researchers. The experiments enabled us to confirm that our method outperforms other methods both in terms of accuracy and processing time.

In the future, we plan to increase the number of behavioral types by including those associated with emergency situations such as kidnapping, etc., for our experiments. In addition, we plan to research a method for enhancing the accuracy of determining emergency situations by combining our vision-based method with other sensor-based methods such as audio sensors.

## Figures and Tables

**Figure 10:**
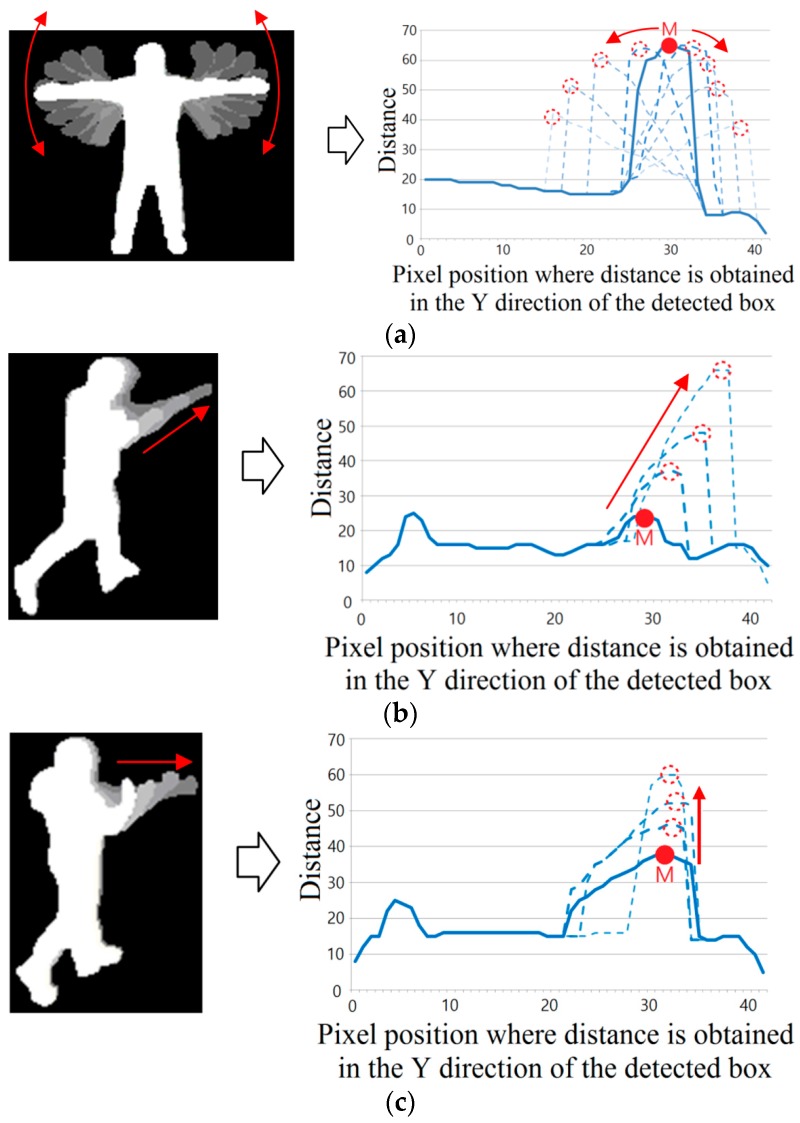
Examples of changes in the position of point M in the profile graph for hand waving and punching. (**a**) Hand waving (the figure on the right shows one profile graph among the two graphs representing the waving of both left and right hands); (**b**) punching (case 1); (**c**) punching (case 2).

**Figure 11:**
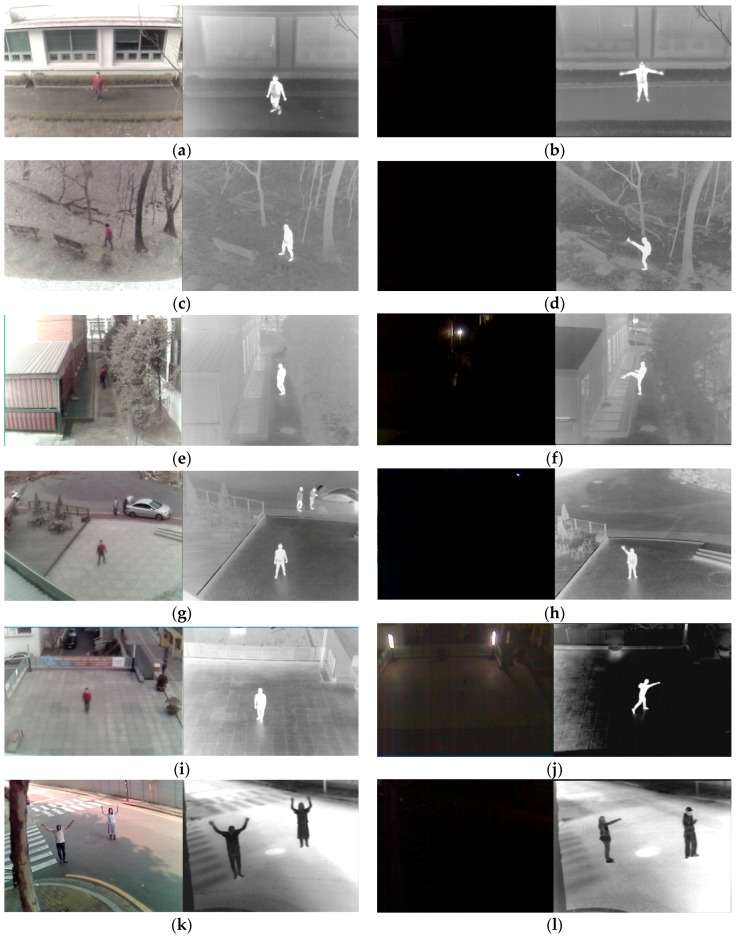
Examples from each of the 11 datasets. (**a**) Dataset I; (**b**) dataset II; (**c**) dataset III; (**d**) dataset IV; (**e**) dataset V; (**f**) dataset VI; (**g**) dataset VII; (**h**) dataset VIII; (**i**) dataset IX; (**j**) dataset X; (**k**) dataset XI; (**l**) dataset XII.

**Table 1:** Decision rules for recognizing the types of behavior in Class 2.

Decision rules	
*Rule 1* (Lying down)	*If* (“*Condition 1* * is TRUE”) : *Lying down* *Else* : *Go to Rule 2*
*Rule 2* (Kicking)	*If* (“M is leg tip” *and* “Condition 2 * is TRUE”) : *Kicking* *Else* : *Go to Rule 3*
*Rule 3* (Hand waving)	*If* (“M is hand tip” *and* “Condition 3 * is TRUE”) : *Hand waving* *Else* : *Go to Rule 4*
*Rule 4* (Punching)	*If* (“M is hand tip” *and* (“Condition 4 * or Condition 5 * is TRUE”)) : *Punching* *Else* : *Go to the rule* (for checking running, walking or undetermined case as shown in the right lower part of Figure 3)

* **Condition 1**: Average *R* of Equation (2) is less than two thresholds (T_r2_ and T_r3_) as shown in Figure 3; * **Condition 2**: both X and Y positions of M point are higher than threshold; * **Condition 3**: The position of M is changed in both X and Y directions in recent N frames as shown in Figure 10a; * **Condition 4**: The position of M is increased in both X and Y directions in recent N frames as shown in Figure 10b; * ***Condition 5***: The position of M is increased only in the Y directions in recent N frames as shown in Figure 10c.

**Table 2:** Description of 12 datasets.

Dataset	Condition	Detail Description
I (see in Figure 11a)	1.2 °C, morning, humidity 73.0%, wind 1.6 m/s	- The intensity of background is influenced by the window of building - Human shadow is reflected on the window, which is detected as another object in thermal image
II (see in Figure 11b)	−1.0 °C, evening, humidity 73.0%, wind 1.5 m/s	- The intensity of background is influenced by the window of building - Object is not seen in visible light image - Human shadow is reflected on window, which is detected as another object in thermal image
III (see in Figure 11c)	1.0 °C, afternoon, cloudy, humidity 50.6%, wind 1.7 m/s	- The intensity of background is influenced by leaves and trees
IV (see in Figure 11d)	−2.0 °C, dark night, humidity 50.6%, wind 1.8 m/s	- The intensity of background is influenced by leaves and trees - Object is not seen in visible light image
V (see in Figure 11e)	14.0 °C, afternoon, sunny, humidity 43.4%, wind 3.1 m/s	- Difference between background and human diminishes because of the high temperature of background
VI (see in Figure 11f)	5.0 °C, dark night, humidity 43.4%, wind 3.1 m/s	- The air heating system of building increases the temperature of part of the building in background - Object is not seen in visible light image
VII (see in Figure 11g)	−6.0 °C, afternoon, cloudy, humidity 39.6%, wind 1.9 m/s	- Halo effect is shown near the human area in thermal image, which makes it difficult to detect the correct human area
VIII (see in Figure 11h)	−10.0 °C, dark night, humidity 39.6%, wind 1.7 m/s	- Halo effect is shown near the human area in thermal image, which makes it difficult to detect the correct human area - Object is not seen in visible light image
IX (see in Figure 11i)	21.9 °C, afternoon, cloudy, humidity 62.6%, wind 1.3 m/s	- Halo effect is shown near the human area in thermal image, which makes it difficult to detect the correct human area - Difference between background and human diminishes due to the high temperature of background
X (see in Figure 11j)	−10.9 °C, dark night, humidity 48.3%, wind 2.0 m/s	- The dataset was collected at night during winter. Therefore, the background in thermal image is too dark because of low temperature - Object is not seen in visible light image
XI (see in Figure 11k)	27.0 °C, afternoon, sunny, humidity 60.0%, wind 1.0 m/s	- Human is darker than road because the temperature of the road is much higher than that of a human in summer - Leg is not clear when kicking behavior happens because the woman in the image wore a long skirt
XII (see in Figure 11l)	20.2 °C, dark night, humidity 58.6%, wind 1.2 m/s	- Human is darker than road because the temperature of the road is much higher than that of a human in summer - Object is not seen in visible light image

**Table 3:** Camera setup used to collect the 11 datasets (unit: meters).

Datasets	Height	Horizontal Distance	Z Distance
Datasets I and II	8	10	12.8
Datasets III and IV	7.7	11	13.4
Datasets V and VI	5	15	15.8
Datasets VII and VIII	10	15	18
Datasets IX and X	10	15	18
Datasets XI and XII	6	11	12.5

**Table 4:** Numbers of frames and the types of behavior in each dataset.

	#Frame		#Behavior	
Behavior	Day	Night	Day	Night
Walking	1504	2378	763	1245
Running	608	2196	269	355
Standing	604	812	584	792
Sitting	418	488	378	468
Approaching	1072	1032	356	354
Leaving	508	558	163	188
Waving with two hands	29588	14090	1752	870
Waving with one hand	24426	15428	1209	885
Punching	21704	13438	1739	1078
Lying down	7728	5488	2621	2022
Kicking	27652	22374	2942	3018
Total	194094		24051	

**Table 5:** Accuracies of behavior recognition by our method (unit: %).

	Day				Night			
Behavior	TPR	PPV	ACC	F_Score	TPR	PPV	ACC	F_Score
Walking	92.6	100	92.7	96.2	98.5	100	98.5	99.2
Running	96.6	100	96.7	98.3	94.6	100	94.7	97.2
Standing	100	100	100	100	97.3	100	97.3	98.6
Sitting	92.5	100	92.5	96.1	96.5	100	96.5	98.2
Approaching	100	100	100	100	100	100	100	100
Leaving	100	100	100	100	100	100	100	100
Waving with two hands	95.9	98.8	99.4	97.3	97.0	99.5	99.6	98.2
Waving with one hand	93.6	99.4	99.3	96.4	90.0	100	99.0	94.7
Punching	87.8	99.5	98.0	93.3	77.4	99.6	96.4	87.1
Lying down	99.4	99.0	98.9	99.2	97.3	98.0	96.3	97.6
Kicking	90.6	95.8	97.2	93.1	88.5	90.3	94.6	89.4
Average	95.4	99.3	97.7	97.3	94.3	98.9	97.5	96.4

**Table 6:** Processing time of our method for each behavior dataset (unit: ms/frame).

	Processing Time	
Behavior	Day	Night
Walking	2.3	2.6
Running	1.5	1.5
Standing	3.2	3.1
Sitting	1.9	2.0
Approaching	3.3	2.9
Leaving	2.9	2.9
Waving with two hands	3.2	3.1
Waving with one hand	2.6	2.1
Punching	2.7	2.3
Lying down	1.2	1.0
Kicking	1.9	2.0
Average	2.4	

**Table 7:** Accuracies of other methods (unit: %) *.

	Fourier Descriptor-Based				GEI-Based				Convexity Defect-Based			
Behavior	TPR	PPV	ACC	F_Score	TPR	PPV	ACC	F_Score	TPR	PPV	ACC	F_Score
Walking	0	0	1.6	-	83.9	97.7	82.4	90.3	17.4	98.4	18.4	29.6
Running	13.0	97.2	16.0	22.9	0	0	3.5	-	23.4	98.4	27.6	37.8
Standing	85.9	100	85.9	92.4	*n/a*	*n/a*	*n/a*	*n/a*	*n/a*	*n/a*	*n/a*	*n/a*
Sitting	59.7	100	59.7	74.8	*n/a*	*n/a*	*n/a*	*n/a*	*n/a*	*n/a*	*n/a*	*n/a*
Waving with two hands	81.4	13.7	47.8	23.5	96.5	19.1	59.6	31.9	89.0	68.6	94.9	77.4
Waving with one hand	78.2	13.8	50.3	23.5	21.2	10.1	73.8	13.7	34.9	73.4	92.4	47.3
Punching	27.1	20.3	74.6	23.2	62.2	16.5	50.1	26.1	39.7	55.7	86.9	46.4
Lying down	12.9	72.0	38.6	21.9	*n/a*	*n/a*	*n/a*	*n/a*	*n/a*	*n/a*	*n/a*	*n/a*
Kicking	61.1	42.0	78.7	49.8	24.7	80.7	86.0	37.8	29.3	47.6	82.2	36.3
Average	46.6	51.0	50.4	48.7	55.5	46.3	65.1	50.5	47.7	77.4	71.8	59.0

* n/a represents “not available” (the method was unable to produce a result for behavior recognition).

**Table 8:** Confusion matrix of the results of behavior recognition by our method (unit: %).

	Predicted	Walking	Running	Standing	Sitting	Waving with two hands	Waving with one hand	Lying down	Kicking	Punching
Actual										
Walking		95.5		0.3	0.4				0.5	
Running		0.4	95.6				0.3		3.3	0.4
Standing				98.5						
Sitting				0.5	94.6					
Waving with two hands						96.4			0.3	
Waving with one hand				0.2	0.1		91.8			0.1
Lying down		0.3			0.6			98.3		
Kicking		0.2			3.0		0.3		89.5	
Punching				0.3	1.6				0.3	82.5

**Table 9:** Summarized comparisons of accuracies and processing time obtained by previous methods and by our method.

Method	TPR (%)	PPV (%)	ACC (%)	F_score (%)	Processing Time (ms/frame)
Fourier descriptor-based	46.6	51.0	50.4	48.7	16.1
GEI-based	55.5	46.3	65.1	50.5	4.9
Convexity defect-based	47.7	77.4	71.8	59.0	5.2
Our method	94.8	99.1	97.6	96.8	2.4
